# Research on Evaluation of Meteorological Disaster Governance Capabilities in Mainland China Based on Generalized λ-Shapley Choquet Integral

**DOI:** 10.3390/ijerph18084015

**Published:** 2021-04-12

**Authors:** Yajun Wang, Fang Xiao, Lijie Zhang, Zaiwu Gong

**Affiliations:** 1 School of Management and Engineering, Nanjing University of Information Science & Technology, Nanjing 210044, China; 20181224009@nuist.edu.cn (Y.W.); lijiezh@163.com (L.Z.); 2 China Meteorological Administration Institute for Development and Program Design, Beijing 100081, China; xiaof@cma.gov.cn; 3 Business School, Linyi University, Linyi 276000, China

**Keywords:** meteorological disaster governance capacity, multi-attribute evaluation, Choquet integral, generalized Shapley function

## Abstract

According to the United Nations report, climate disasters have intensified in the past 20 years, and China has the largest number of disasters in the world. So the study of meteorological disaster governance capacities is critically important for China. We designed a meteorological disaster governance capacity evaluation system to calculate the evaluation values by using the generalized λ-Shapley Choquet integral, a method that considers the interaction between indicators. We used various official statistical yearbooks and internal data of China Meteorological Administration (CMA) and weight intervals set by meteorologists for each level of indicators to calculate the evaluation values of meteorological disaster governance capacity in mainland provinces, from 2014 to 2018. We compared them with other methods (entropy weight method, Technique for Order Preference by Similarity to an Ideal Solution (TOPSIS), and Analytic Hierarchy Process (AHP)), and the results showed that the results calculated by the designed interaction method provided in this paper are more stable and differentiated. The results show that provincial meteorological disaster governance capacities in Mainland China are characterized by uneven development and a pro-slight polarization phenomenon. This leads to policy recommendations: Provinces should strengthen the construction of meteorological disaster information; provinces with outstanding capacity must strengthen the experience sharing with provinces with lower capacity.

## 1. Introduction

In recent years, drastic climate change and various meteorological disasters have occurred frequently, seriously threatening the survival and development of human beings all over the world. China is located in the middle of two major natural disaster belts globally, namely the Northern Hemisphere Mid-Latitude Disaster Belt and the Pacific Rim Disaster Belt. It is one of the most severely impacted countries in the world by natural disasters. Meteorological disasters account for more than 70% of all-natural disasters occurring in China [1]. The economic losses brought by meteorological disasters account for about 2/3 of the financial losses from natural disasters [2]. China’s annual average direct economic loss caused by meteorological disasters has increased from less than 100 billion yuan in the 1950s to more than 300 billion yuan in the 21st century [3], such as direct economic losses of 503.29 billion yuan in 2016 [4], and financial losses caused by meteorological disasters of up to 318 billion yuan in 2019 [5]. Meanwhile, China’s natural disaster prevention and control capacity are generally weak. It is urgent and essential to improve the governance capacity of natural disasters, primarily meteorological disasters, which is a significant issue related to people’s safety, lives, properties, and national security.

Many scholars have now made many studies on meteorological hazards in China. Most of these papers have accumulated in the areas of individual meteorological hazards, such as floods and droughts [6,7,8], typhoons [9], and other more homogeneous meteorological hazards, or in terms of individual geographical divisions, such as the Yangtze River Delta [10,11], the Maritime Silk Road [9], and Southern China [12]. There are certainly some excellent review articles that finely sort out the meteorological hazard situation in China [13] and explore research methods [14]. However, these studies are often based on one or a few meteorological hazards or limited to a particular geographical area. In contrast, very few studies have been conducted on meteorological disaster governance capacity from Mainland China or have explored the concept of governance. Nowadays, China is paying more and more attention to improving and strengthening meteorological disaster governance capacity, and higher requirements have been put forward in disaster emergency response [15]. Exploring the meteorological disaster governance capacity in Mainland China from a national perspective is the theoretical starting point of this paper and the starting point of this paper in collaboration with the China Meteorological Administration. Meteorological disaster governance capacity is a comprehensive capacity and we often use multi-attribute decision-making (MADM) in studying it with these attributes. Therefore, in this paper, we establish the evaluation model of meteorological disaster governance capability, considering the interaction of attributes to study the meteorological disaster governance capability.

This paper establishes a meteorological disaster governance capacity assessment index system, in which the data are obtained from significant yearbooks (“China Statistical Yearbook”, “Yearbook of Meteorological Disasters in China”, “China Civil Affair Statistical Yearbook—Statistics of China Social Services”, and “China Agriculture Yearbook”) and the data of meteorological modernization indicators given by the Bureau of Meteorology. The setting of exact weights is not easy, and therefore, in this paper, the interval assignment of weights is adopted, and we set weight intervals for each indicator based on experts’ experience in each meteorological field. The fuzzy multi-attribute decision evaluation method was then used to derive each provincial area’s evaluation results [16,17]. However, the simple weighted average cannot capture the indicators’ redundancy and synergy [18,19]. When evaluating meteorological disaster governance capacity using fuzzy multi-attribute decision-making methods, it cannot merely be assumed that the attributes are independent of each other. Many scholars have agreed that the assumption of independence between attributes is inappropriate in decision-making problems [20,21,22]. Therefore, this paper proposes a multi-attribute evaluation method that considers the interaction between attributes to evaluate each province’s meteorological disaster governance capacity. Moreover, this paper considers the case that the attribute weights are not entirely known, uses Shapley integral and λ-fuzzy measure to reduce the difficulty of solving the fuzzy measure problem, and finally uses the Choquet integral to derive the final evaluation value [23,24]. Fuzzy measures give up additivity compared to traditional probability measures, so they have a more comprehensive range of monotonicity and are more consistent with human inference when solving problems [25,26,27]. The Choquet integral proposed by the French scientist Choquet can deal well with the issue of correlations existing between attributes in comprehensive evaluation problems [28] and is widely used in the field of multi-attribute decision-making [29,30].

In Section 2, we construct a meteorological disaster governance capacity indicator system. In Section 3, we build a meteorological disaster governance model, considering the interaction of attributes. In Section 4, the indicator data and the weight intervals given by the experts’ experience are brought into the model to calculate the results, and the results are compared with entropy weight method, Technique for Order Preference by Similarity to an Ideal Solution (TOPSIS), and Analytic Hierarchy Process (AHP) methods. In Section 5, we analyze the results and propose corresponding policy recommendations.

## 2. Meteorological Disaster Governance Capacity and Index System Construction

### 2.1. Analysis of Meteorological Disaster Governance Capacity

Meteorological disasters have the characteristics of general natural disasters, i.e., the damage caused varies according to the amount and speed of material and energy movement; the topography of the region and other factors can cause the amount and intensity of material and energy movement to change in different locations in the area; various disaster-bearing bodies have additional resilience to different materials and energy, and the length of the disaster process is related to the level of post-disaster recovery. According to the essential elements of catastrophe, we can summarize the key to reduce disaster losses. That is, disaster prevention infrastructure, construction planning or construction input based on careful consideration of disaster prediction, regional topography, and characteristics of disaster-bearing bodies for several years in the future; rapid and accurate disaster risk zoning based on sudden meteorological disaster prediction, topography, and other factors; precise disaster risk-reduction suggestions; and establishment of the development-oriented disaster recovery system.

In recent years, China has actively pushed governments at all levels to improve their disaster governance capabilities. In his report to the 19th Party Congress, General Secretary Xi Jinping proposed to “enhance disaster prevention, mitigation and relief capabilities”, and has repeatedly given essential instructions on “scientific and accurate forecasting”, “implementing responsibilities, improving systems, integrating resources, and coordinating efforts to comprehensively improve national comprehensive disaster prevention, mitigation and relief capabilities”. In 2017, the China Meteorological Administration (CMA) issued the “Opinions of CMA on Strengthening Meteorological Disaster Prevention, Mitigation and Relief”, which proposed “two persistent and three transformations” as the goal of disaster governance [31].

### 2.2. Rating Index System Construction

In this paper, based on the characteristics and specificity of meteorological disasters, we consider the whole process of disaster governance [32,33] in conjunction with the key of disaster loss and divide meteorological disaster governance capabilities into three major categories of capabilities from the time and resource dimensions of emergency management (including the three processes of efficient prevention, timely response, and orderly recovery), as well as from the new requirements put forward by the state for disaster governance. These three categories of capabilities are then divided into ten governance capabilities, refined into 25 specific indicators, as shown in Table 1.

Next, we give a detailed explanation of Table 1. We divide the description into three parts by the primary indicator:

(1) Legal construction capacity (c1) mainly focuses on constructing laws and regulations related to meteorological disasters in each local area, including the annual promulgation of standards about meteorological disaster defense, the coverage rate of meteorological disaster defense planning and the completeness rate of meteorological disaster emergency plans. The construction capacity of defense mechanisms mainly covers constructing mechanisms related to meteorological disasters, including the total number of comprehensive disaster reduction demonstration zones and the number of new ones added annually and the utilization of large-scale climate resource development projects. Currently, the capacity of defense engineering construction only has a three-level indicator of disaster relief reserve units’ total storage capacity. All three capabilities are construction and planning to cope with possible future meteorological disasters, summarized as law and building defense capability.

(2) The disaster detection capability (c2) focuses on the completeness and density of monitoring facilities, mainly examining the number of weather radar observation stations, the number of automatic weather stations, the number of lightning location detection stations and the number of satellite-data-receiving stations in each region, which are the four tertiary indicators. The disaster prediction capability targets the prediction aspect, mainly examining the accuracy of these monitoring facilities on various types of weather conditions, including the accuracy of 24-h precise rain forecasts, the advance of strong convective weather warnings, and the accuracy of heavy rainfall warnings, which are three tertiary indicators. The ability to transmit early warning information is mainly examined in the three tertiary hands of weather warning information in social units, information broadcasting media and social institutions. The disaster-related information acquisition capability focuses on the three three-level indicators: the two-way sharing rate of the meteorological information department and the deployment rate of village (community) township (street) meteorological informants and coordinators. These four capabilities are also essential elements of the meteorological modernization index system, which can be used to obtain and process information in meteorological disaster governance, the basis for accurate prediction of meteorological disasters before the disasters, and the basis for precise decision-making before tragedies occur.

(3) The remaining three secondary indicators mainly reflect timely response in disasters and post-disaster relief. The social resource mobilization capacity includes two tertiary indicators of insurance premium income and welfare lottery fund expenditure. The capacity of proactive disaster risk reduction mainly examines the reserve of meteorological disaster mitigation equipment, including two three-level indicators of available anti-aircraft guns and available rockets. Disaster relief capacity examines the medical and health services, medical and health expenditures, and the provision of shelters that the government and society can provide after a disaster. The secondary indicators of disaster relief security capacity include three tertiary indicators: the number of institutional beds per 10,000 people, local financial expenditure on health care and local per capita (10,000 yuan) expenditure on general public services. These three capacities can be summarized as disaster relief capacity (c3).

These ten indicators cover the pre-disaster, disaster and post-disaster phases of meteorological disasters and focus on the government’s pre-disaster meteorological disaster prevention capability and information technology construction, which are the requirements of the current national goal of “two insistences and three changes” in disaster governance. Meanwhile, the meteorological disaster governance capability is divided into three essential items: creating the evaluation index system. Then the three important primary indicators are refined into ten secondary indicators. The ten secondary indicators are then refined into 25 tertiary indicators, following the requirements of scientific, feasibility, hierarchy, flexibility, and dynamism of the index system.

## 3. Models and Computational Methods

### 3.1. Fuzzy Measure and Choquet Integral

**Definition** **1.** *A fuzzy measure on a finite set*−N*is a real-valued set function defined on the powerset of N, i.e.,*$\;\mu :P\left(N\right)\to \left[0,1\right]$*, satisfying the following*:

(1) $\mu \left(\emptyset \right)=0,\mu \left(N\right)=1$;
(2) If A,B∈P(N) and $A\subseteq B,\mu \left(A\right)\leq \mu \left(B\right)$.

**Definition** **2.** *Let*$X=\left\{x_{1},x_{2},\ldots ,x_{n}\right\},\;f$*be a non-negative real-valued function defined on*X,*and*μ*be a fuzzy measure on*N. *The Choquet integral of the function*f*is defined as follows*:

(1) $$C_{\mu }\left(f\left(x_{1}\right),f\left(x_{2}\right),\ldots ,f\left(x_{n}\right)\right)=\overset{n}{\underset{i=1}{\sum }}f\left(x_{i}\right)\left(\mu \left(A_{i}\right)-\mu \left(A_{i+1}\right)\right)$$

*where*$f\left(x_{1}\right)\leq f\left(x_{2}\right)\leq \ldots \leq f\left(x_{n}\right)\;,A_{i}=\left\{x_{i}\ldots x_{n}\right\}\;,A_{n+1}=\;\emptyset$.

Since the fuzzy measure is defined on the power set of a set, the number of variables in the fuzzy measure increases exponentially with the cardinality of the set. To reduce the complexity of solving and computing fuzzy measures while reflecting the interaction between evaluation indicators, Sugeno [34] proposed the λ-fuzzy measure.

**Definition** **3.** *For any*A,B⊆N,A∩B=∅. *If the fuzzy measure*$g_{\lambda }$*satisfies the following conditions:*

(2) $$g_{\lambda }\left(A\cup B\right)=g_{\lambda }\left(A\right)+g_{\lambda }\left(B\right)+\lambda g_{\lambda }\left(A\right)g_{\lambda }\left(B\right)$$

*where*$\lambda \in \left(-1,\infty \right)$*, and then*$g_{\lambda }$*is said to be a*λ*-fuzzy measure.*

From Definition 3, if  λ > 0, $g_{\lambda }$ is said to be a superadditive measure, indicating some complementary interaction between coalitions *A* and *B*. If −1 < λ < 0, then $g_{\lambda }$ is said to be a subadditive measure, indicating redundant interactions between coalition *A* and coalition *B*.

(3) $$g_{\lambda }\left(A\right)=\left\{\begin{array}{ll} \frac{1}{\lambda }\left(\underset{i\in A}{\prod }\left[1+\lambda g_{\lambda }\left(i\right)\right]-1\right), & \lambda \neq 0\\ \underset{i\in A}{\sum }g_{\lambda }\left(i\right), & \lambda =0 \end{array}\right.$$

From $\left(\mu ,N\right)=1$, the value of λ can be determined by Equation (5), so when the value of $g_{\lambda }\left(i\right)$ is given, the value of λ can be calculated. From Equation (4), for a set N with *n* elements, only *n* values need to be determined to obtain the fuzzy measure of any subset of the set N. If $\overset{n}{\underset{i\in A}{\sum }}g_{\lambda }\left(i\right)=1$, then λ = 0.

(4) $$\lambda +1=\underset{i\in N}{\prod }\left[1+\lambda g_{\lambda }\left(i\right)\right]$$

#### 3.1.1. Determination of Attribute Weights

Since we cannot give each indicator a substantial weight, we get only partial weights more often than not. In this paper, we consider the interaction between the attributes by utilizing the Shapley value to build a mathematical model and then find the optimal fuzzy measure on the set of attributes. The Shapley function is one of the most critical allocation indicators in cooperative games, denoted as follows:

(5) $$\phi_{i}\left(\mu \right)\;=\underset{\begin{array}{c} S\subseteq N\backslash i \end{array}}{\sum }\frac{\left(n-s-1\right)!s!}{n!}\left(\mu \left(S\cup i\right)-\mu \left(S\right)\right)\;,\;\forall \;i\in N$$

#### 3.1.2. Generalized λ-Shapley Choquet Integral

The Shapley value can be applied to multi-attribute decision-making to express the importance coefficient of each indicator [35], which is defined by the equation:

(6) $$\varphi_{s}^{sh}\left(\mu ,N\right)=\underset{\begin{array}{c} T\subseteq N\backslash S \end{array}}{\sum }\frac{\left(n-s-t\right)!t!}{\left(n-s+1\right)!}\left(\mu \left(S\cup T\right)-\mu \left(T\right)\right),\;\forall S\subseteq N$$

where μ is a fuzzy measure on N.

We consider the importance and relevance of the combination of elements globally according to the generalized λ-Shapley Choquet integral defined by Meng [21]. The λ-fuzzy measure on the set N is first given, denoted as:

(7) $$\varphi_{s}^{sh}\left(g_{\lambda },N\right)=\underset{\begin{array}{c} T\subseteq N\backslash S \end{array}}{\sum }\frac{\left(n-s-t\right)!t!}{\left(n-s+1\right)!}\left(g_{\lambda }\left(S\cup T\right)-g_{\lambda }\left(T\right)\right),\;\forall S\subseteq N\;$$

From Equation (4), if $S=\left\{i\right\}$, then we have the following:

(8) $$\varphi_{i}^{sh}\left(g_{\lambda },N\right)=\underset{\begin{array}{c} S\subseteq N\backslash i \end{array}}{\sum }\frac{\left(n-s-1\right)!s!}{n!}g_{\lambda }\left(i\right)\underset{j\in S}{\prod }\left[1+\lambda g_{\lambda }\left(j\right)\right],\;\forall i\subseteq N$$

Combining with Definition 2, we define the arithmetic generalized λ-Shapley Choquet integral formula [23] as follows:

$$C_{\varphi^{sh}\left(g_{\lambda },N\right)}\left(f\left(x_{\left(1\right)}\right),f\left(x_{\left(2\right)}\right),\ldots ,f\left(x_{\left(n\right)}\right)\right)$$

(9) $$={⊕}_{i=1}^{n}\left(\varphi_{A_{\left(i\right)}}^{sh}\left(g_{\lambda },N\right)-\varphi_{A_{\left(i+1\right)}}^{sh}\left(g_{\lambda },N\right)\right)f\left(x_{\left(i\right)}\right)$$

where $f\left(x_{1}\right)\leq f\left(x_{2}\right)\leq \ldots \leq f\left(x_{n}\right),A_{\left(i\right)}=\left\{x_{\left(i\right)}\ldots x_{\left(n\right)}\right\},A_{\left(n+1\right)}=\;\emptyset$.

### 3.2. Meteorological Disaster Governance Capability Evaluation Method Based on λ-Shapley Choquet Integral

The multi-attribute evaluation method used in this paper considers the interaction between attributes. When the weights of the attributes are unknown or partially known, the weights of the attributes are determined first, and then the capability evaluation values are calculated.

The optimal fuzzy measurement model on the attribute set *C* is established as follows:

$$min\overset{m}{\underset{i=1}{\sum }}\overset{n}{\underset{j=1}{\sum }}d_{ij}\Phi_{c_{j}}\left(v,C\right)$$

(10) $$s.t.\left\{\begin{array}{l} v\left(C\right)=1\\ v\left(S\right)\leq v\left(T\right),\;\forall S,T\subseteq C,s.t.\;\;S\subseteq T\\ v\left(c_{j}\right)\in U_{c_{j}}\;,\;v\left(c_{j}\right)\geq 0\;,\forall c_{j}\in C \end{array}\right.$$

where $\Phi_{c_{j}}\left(v,C\right)$ is the Shapley value of $c_{j}$, v is a fuzzy measure on C, and $U_{c_{j}}$ is the range of values of $c_{j}$.

The $d_{ij}$ in the model is replaced by the original TOPSIS value in this paper. The calculation steps of the original TOPSIS value are rough, as follows:

(1) Normalize the original data. All the data in this paper are benefit-based indicators, so they do not need to be normalized. For cost-based indicators, they need to be normalized.

(2) Use the maximum value of each indicator as to the positive ideal solution $Z_{j}^{+}$ and the minimum value as the negative ideal solution $Z_{j}^{-}$, and then find the distance $D_{i}^{+}$ and $D_{i}^{-}$ for each evaluation value and positive ideal solution and negative ideal solution.

$$D_{i}^{+}=\sqrt{\sum_{j=1}^{m}{\left(Z_{j}^{+}-Z_{ij}\right)}^{2}},\;D_{i}^{-}=\sqrt{\sum_{j=1}^{m}{\left(Z_{j}^{-}-Z_{ij}\right)}^{2}}$$

(3) Calculate the integrated value of the ith evaluation object:$\;d_{ij}=\frac{D_{i}^{+}}{D_{i}^{+}+D_{i}^{-}}$.

Because the original TOPSIS does not need to add weights when calculating positive and negative ideal solutions, this paper uses the Shapley value $\Phi_{c_{j}}\left(v,C\right)$ as weights in the Model (10).

The steps for evaluating the meteorological disaster governance capacity of provinces in Mainland China with incomplete weight information and interactions between 2014–2018 are given below:

**Step 1:** Using the optimal fuzzy measure linear programming Model (10) to find the fuzzy measure of the sub-criteria level, the fuzzy measure $v\left(c_{jk}\right)$ is expressed by $g\left(c_{jk}\right)$ and brought into Equation (4) to solve for λ. Then, the λ-fuzzy measure is calculated according to Equation (3), and the generalized Shapley value of the tertiary index is found using Equations (7) and (8).

**Step 2:** The generalized λ-Shapley Choquet integral formula is used to calculate the third-level indicators’ data to obtain the second-level indicators’ evaluation value.

**Step 3:** As in Step 1, the generalized Shapley values of the second-level indicators are calculated by bringing the second-level indicators into the Model (10), and then, as in Step 2, the first-level indicator values are calculated.

**Step 4:** Similar to Step 3, the comprehensive evaluation value is calculated.

**Step 5:** Finally, repeat Steps 1–4 to calculate each province’s comprehensive evaluation value from 2014 to 2018.

## 4. Evaluation Value Calculation and Method Comparison

### 4.1. Evaluation of Provincial Meteorological Disaster Governance Capacity in Mainland China

In this paper’s indicator system, the data of the three-level indicators are obtained from significant yearbooks (“China Statistical Yearbook”, “Yearbook of Meteorological Disasters in China”, “China Civil Affair Statistical Yearbook—Statistics of China Social Services”, and “China Agriculture Yearbook”) and the data of meteorological modernization indicators given by the Bureau of Meteorology. Based on the interval weights drawn up by experts for each level of indicators, the provinces’ meteorological disaster governance capacity in Mainland China from 2014 to 2018 was then evaluated by the model presented in Section 3. The meteorological experts’ weight intervals for the indicators given for each level are shown in Table 2.

Using the data of 2014 as an example, perform the calculation as follows:

**Step 1:** Following Model (10), the optimal fuzzy measure linear programming model on the attribute set $c_{11}$ is obtained, as shown below:

$$min\;2.730\left(v\left(c_{111}\right)-v\left(c_{112},c_{113}\right)\right)-3.504\left(v\left(c_{112}\right)-v\left(c_{111},c_{113}\right)\right)+0.773\left(v\left(c_{113}\right)-v\left(c_{111},c_{112}\right)\right)+18.085$$

$$s.t.\left\{\begin{array}{c} v\left(c_{111},c_{112},c_{113}\right)=1\;\\ v\left(S\right)\leq v\left(T\right),\;\forall S,T\subseteq \left\{c_{111},c_{112},c_{113}\right\},\;S\subseteq T\\ v\left(c_{11}\right)=\left[0.5,0.7\right]\;\\ v\left(c_{12}\right)=\left[0.25,0.4\right]\;\\ v\left(c_{13}\right)=\left[0.25,0.4\right]\; \end{array}\right.$$

By solving the model, we get the following:

$$v\left(c_{111}\right)=\;v\left(c_{111},c_{113}\right)=0.5,$$

$$v\left(c_{112}\right)=0.4,\;v\left(c_{113}\right)=0.25,$$

$$v\left(c_{111},c_{112}\right)=v\left(c_{112},c_{113}\right)=\;v\left(c_{111},c_{112},c_{113}\right)=1.$$

The fuzzy measure $v\left(c_{jkl}\right)$ is expressed by $g\left(c_{jkl}\right)$ and brought into Equation (4) to solve for λ = −0.369. Calculating the λ-fuzzy measure according to Equation (3) yields.

$$g\left(c_{111}\right)=\;0.5,\;g\left(c_{112}\right)=0.4,\;g\left(c_{113}\right)=0.25,$$

$$g\left(c_{111},c_{112}\right)=0.826,\;g\left(c_{111},c_{113}\right)=0.704,$$

$$g\left(c_{112},c_{113}\right)=0.613,\;g\left(c_{111},c_{112},c_{113}\right)=1.$$

From Equations (7) and (8), we have the following:

$$\varphi_{\emptyset }^{sh}\left(g_{\lambda },C\right)=0,\;\;\varphi_{c_{111}}^{sh}\left(g_{\lambda },C\right)=0.442,\;\;\varphi_{c_{112}}^{sh}\left(g_{\lambda },C\right)=0.347,$$

$$\varphi_{c_{113}}^{sh}\left(g_{\lambda },C\right)=0.211,\;\varphi_{\left\{c_{111},c_{112}\right\}}^{sh}\left(g_{\lambda },C\right)=0.788,\;\;\varphi_{\left\{c_{111},c_{113}\right\}}^{sh}\left(g_{\lambda },C\right)=0.652,$$

$$\varphi_{\left\{c_{112},c_{113}\right\}}^{sh}\left(g_{\lambda },C\right)=0.557,\;\varphi_{\left\{c_{111},c_{112},c_{113}\right\}}^{sh}\left(g_{\lambda },C\right)=1.$$

Similarly, the generalized Shapley values of the remaining tertiary indicators can be obtained, as shown in Table 3.

**Step 2**: Rearrange the evaluation values from smallest to largest to get $c_{111}^{Beijing}≺c_{113}^{Beijing}≺c_{112}^{Beijing}$, and use the generalized λ-Shapley Choquet integral Equation (9) to get the composite value of Beijing’s attribute $c_{11}$ as $F_{C_{11}}^{Beijing}=0.0304$. Similarly, we can get the comprehensive evaluation values of Beijing’s attributes $c_{12}$ and $c_{13}$ as $F_{C_{12}}^{Beijing}=0.0380,F_{C_{13}}^{Beijing}=0.0062$. The same applies to other secondary indicators, which are brought into Model (10), to obtain the secondary indicator evaluation values, as shown in Table 4.

**Step 3**: The data in Table 4 are brought into Model (10) to obtain the secondary indicators’ generalized Shapley values, as shown in Table 5.

**Step 4**: Similarly, the integrated evaluation value for 2014 was found, and the results are shown in Table 6.

**Step 5:** Repeat Steps 1–4 to calculate the comprehensive evaluation value (CEV) for each province, from 2014–2018, as shown in Table 7.

**Figure 1:**
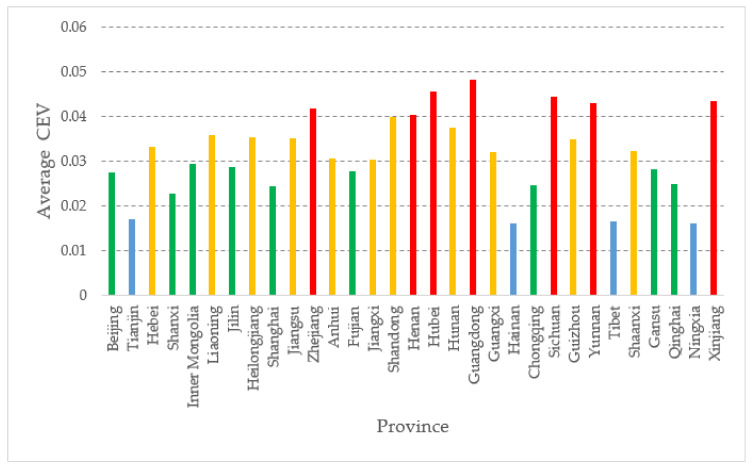
Average CEV by province.

From Table 7, we can see that the comprehensive evaluation value (CEV) of each province does not change much from 2014 to 2018. So, in order to make the graph look more concise and easy to understand, we take the average of the comprehensive evaluation value from 2014 to 2018 and continue to make the graph, as shown in Figure 1.

From Figure 1, we can see that the average comprehensive evaluation value of 31 provinces in Mainland China from 2014 to 2018 can be divided into four levels, which are greater than or equal to 0.04 (first level, red histogram), between 0.03 and 0.04 (second level, yellow histogram), between 0.02 and 0.03 (third level, green histogram), between 0.01 and 0.02 (fourth rank, blue histogram). There are 7 provinces in the first rank (Zhejiang, Henan, Hubei, Guangdong, Sichuan, Yunnan, and Xinjiang); 11 provinces in the second rank (Hebei, Liaoning, Heilongjiang, Jiangsu, Anhui, Jiangxi, Shandong, Hunan, Guangxi, Guizhou, and Shaanxi); 9 provinces in the third rank (Beijing, Shanxi, Inner Mongolia, Jilin, Shanghai, Fujian, Chongqing, Gansu, and Qinghai); and 4 provinces in the fourth rank (Tianjin, Hainan, Tibet and Ningxia). This result is basically in line with the reality that the top-ranked provinces are experienced in disaster management capacity.

### 4.2. Comparison of Methods

The model used in this paper has some similarity with the entropy weight TOPSIS model, so the same data are used and brought into the entropy-weighted TOPSIS model. Moreover, the superiority of the model developed in this paper considering the interaction is discussed in terms of both the maximum ordinal difference and the weights.

#### 4.2.1. Comparison with Entropy Method TOPSIS Model

Using the same data, we calculated the weights of the 25 indicators from 2014 to 2018 using the entropy weight method as shown in Table 8.

From Table 8 we can obviously see that the weights of some indicators, such as c233, c321, and c322, are so small as to be almost negligible. The entropy weight method obtains the weights entirely from the dispersion degree of objective data, i.e., the size of entropy. While entropy is used to measure uncertainty, the greater the dispersion of the indicator (the greater the uncertainty) the greater the entropy value, indicating that the more information the indicator value provides, the greater the weight of the indicator should be. Moreover, some indicators do not vary much nationally, so the weights are not high, but the importance of these indicators may be high. For example, some indicators under information-processing capacity (c2) have a very low weight close to 0, but in real life, they are not less important.

**Figure 2:**
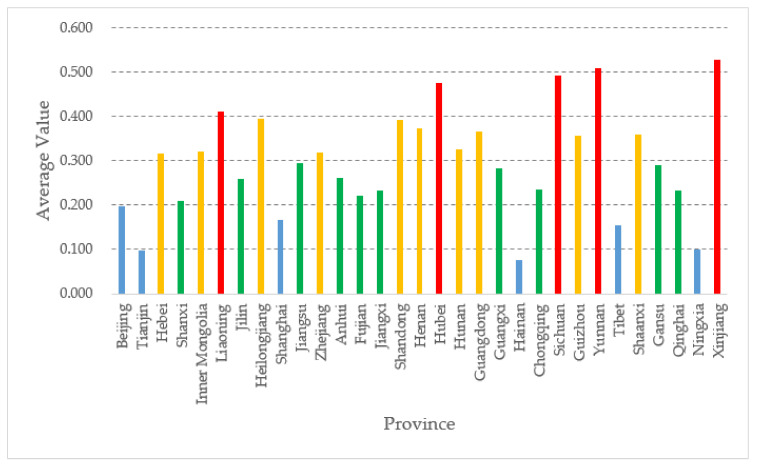
Average CEV by province, entropy weight TOPSIS model.

We then calculated the meteorological disaster governance capacity of 31 provinces, from 2014 to 2018, by using the entropy-weight method to calculate the weights by ourselves, combined with the TOPSIS model. We then took the average and made the graph shown in Figure 2:

As can be seen from Figure 2, there are some differences from the results in Figure 1 because of the influence of the weight defined by the entropy weight method. The main difference lies in the low scores of the four municipalities, which are all classified in the lowest rank, which obviously lacks rationality. The reason for this phenomenon is that the entropy weight method calculates the weights according to the variability of the information contained in each indicator in order to determine the indicator weights, relying only on the discrete degree of the data itself. Entropy is used to measure uncertainty, and the greater the dispersion of the indicator (the greater the uncertainty), the greater the entropy value, indicating that the more information the indicator value provides, the greater the weight of the indicator should be. Moreover, some indicators do not vary much nationally, so the weight is not high, but the importance of these indicators may be high, such as some indicators under information-processing capacity. Moreover, four municipalities directly under the central government, such as Beijing, have higher population density, so they will have lower values for some indicators involving per capita, so they yield poorer results. Therefore, in both models, the composite scores of the four municipalities are not high, and the model considering interaction performs better than the entropy weight TOPSIS method. However, how to estimate the meteorological disaster governance capacity of the four municipalities directly under the central government more accurately is a work that needs to be accomplished in the future.

Analytic Hierarchy Process (AHP) is a combined qualitative and quantitative decision analysis method, by judging the relative importance of each measurement index, and then obtaining the weight of each index in the decision scheme; entropy weighting method is a data-based weighting method, while hierarchical analysis method is an empirical weight method. The method in this paper requires less qualitative judgment than the hierarchical analysis method (only the experts need to give the weight intervals of each indicator in the hierarchy) and combines objective data, which has higher operability and is a combined qualitative and predetermined quantitative method, and uses the generalized λ-Shapley function to remove the interaction between indicators, and the weights obtained under this method are also purer.

#### 4.2.2. Comparison of Maximum Sequence Differences

**Figure 3:**
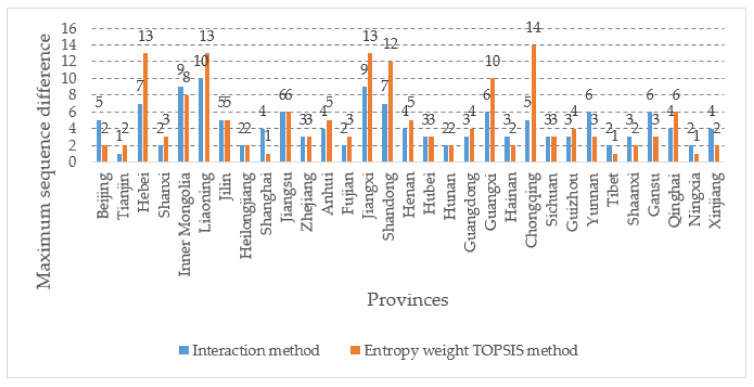
The maximum sequence difference under the two methods.

After we calculated the composite evaluation value of 31 provinces from 2014 to 2018, using two methods, the provinces were ranked by the composite value, and then the difference between the highest and lowest rank of each province was taken as the maximum ordinal difference, and a histogram was made as shown in Figure 3:

From Figure 3, we can see that the results calculated by the entropy weight TOPSIS method and the results calculated by the evaluation method used in this paper considering interaction differ in the maximum ordinal difference, especially in the five provinces of Hebei, Liaoning, Jiangxi, Shandong, Guangxi, and Chongqing, where the difference is enormous. We know that the larger the ordinal difference is, the less stable it is, and the improvement of disaster management capacity is a long-term process, and it is more difficult to have results with such a significant jump as those five that provinces, in contrast, the calculation results of this paper’s model are more reasonable.

#### 4.2.3. Comparison of Weights

We consider the specificity of the method in this paper: Only the weights of the first level indicators are extracted, and a comparison is made between the weights of the first level indicators and the weights derived from the entropy-weight method, as shown in Table 9.

From Table 9, we can see that the distribution of the weights of the entropy TOPSIS method is that c3 accounts for more than 40%, and c1 and c2 are similar. Meanwhile, in the results of the model designed in this paper, the three indicators are almost equally divided in 2014 and 2015, c2 occupies more than 50% of the weight from 2016 to 2018, and the remaining part is equally divided by c1 and c3. Moreover, just in 2016, the China Meteorological Administration proposed in the modernization of meteorology, the goal of informatization and big data in the future development of meteorology [36], and the indicator of c2 information-processing capacity has increased in proportion from 2016 is very reflective of the fact. However, the weight obtained by the entropy weight method in the c2 indicator is not high, and even in some years, it is at the lowest of the three, which reflects the superiority of the method in this paper.

## 5. Conclusion and Policy Recommendations

### 5.1. Conclusions

In this paper, we devote ourselves to studying meteorological disaster governance capacity and evaluating the meteorological disaster governance capacity of provinces in Mainland China, using multi-attribute decision-making. When we assess each local area’s meteorological disaster governance capacity, we use the generalized λ-Shapley Choquet integral to eliminate the interaction between attributes. We build an evaluation model considering the interaction of attributes and then substitute the data and expert weight intervals to calculate the comprehensive evaluation value of meteorological disaster governance capacity of Mainland China’s provincial areas from 2014 to 2018. Finally, the calculation results of this paper and the model are compared with other MADM models (entropy weight method, Technique for Order Preference by Similarity to an Ideal Solution(TOPSIS), and Analytic Hierarchy Process (AHP)). The results of this paper are more stable and reasonable.

According to Figure 1, the development of meteorological disaster governance capacity is unbalanced among provinces in Mainland China, with few provinces having a strong capacity and most of them in the middle rank. The top-ranked provinces, especially Guangdong, Sichuan, and Hubei, pay special attention to meteorological disaster governance and information technology construction, which can be seen from these local governments’ official websites. These provinces have mastered the technology of meteorological disaster management and have entered a virtuous cycle. Except for the four municipalities directly under the central government, the provinces with weaker capacity, which are the blue provinces in the chart, are Tibet, Ningxia, Hainan, and Tianjin. Except for Tianjin, the rest of these places are areas in China with harsh natural environmental conditions and relatively backward economic levels. Understandably, the meteorological disaster governance capacity is weak. By comparing with some articles of the same type [9,10], this paper’s conclusions have some similarities. For example, the two provinces of Zhejiang and Guangzhou are more vital in meteorological disaster management, while Fujian and Hainan have lower evaluation values. In terms of methodological use, this paper uses the MADM method to measure the meteorological hazard management capacity of provinces in Mainland China, which has been used many times in the study of meteorological hazards [14], and this paper also takes into account the interactivity, and the results obtained are more stable and reasonable than the standard MADM method.

### 5.2. Policy Recommendations

Based on the findings indicated in the above conclusion, we can make the following policy recommendations:

(1) Strengthen the informatization of meteorological disaster governance: In 2016, the China Meteorological Administration proposed informatization and big data in future meteorological development in meteorology modernization. The informatization of meteorological disaster governance is also the consensus of governments worldwide [37]. Modernized meteorological disaster governance requires the support of big data, cloud computing, and other data-processing technologies. More precise data analysis and processing can strengthen meteorological disaster prediction accuracy and monitoring, the software part of the meteorological disaster management information construction. The hardware part is that each place should reasonably invest in central weather disaster monitoring and prediction equipment and the recruitment of relevant high-tech talents.

(2) Experience sharing: As shown in Figure 1, the provinces with high capacity are getting better and better, and there is a trend of polarization, but this is uneven and unhealthy. The valuable experience of meteorological disaster governance needs to be shared, especially by the provinces that perform relatively poorly. China’s meteorological disaster governance has received considerable attention in recent years, and experience sharing is needed to improve nationwide. It can be seen from the zoning map that the provinces with lower and higher scores are relatively unchanged, especially those in the last echelon. So it is possible to set up key help targets and focus on the provinces with lower scores. Furthermore, provinces with higher scores in meteorological disaster governance capacity can be tested and shared with neighboring provinces, which is done by considering the similarity of natural environment in neighboring provinces.

## Figures and Tables

**Table 1:** Meteorological disaster governance capacity evaluation index system.

Primary Indicator	Secondary Indicator	Tertiary Indicator
Law and building defense capacity c1	Legal system building capacity c11	Legal local meteorological disaster standard cumulative count c111
		Meteorological disaster defense planning provincial, municipal, and county coverage rate—city c112
		Completion rate of meteorological disaster emergency plans c113
	Defense mechanism construction capacity c12	Number of comprehensive disaster reduction demonstration communities c121
	Disaster defense engineering construction capacity c13	Total storage capacity c131
Information-processing capacity c2	meteorological disaster monitoring capacity c21	Number of operational weather radar observation stations (pcs) c211
		Number of automatic weather stations (one) c212
		Number of operational stations for lightning-positioning monitoring (pcs) c213
		Satellite data receiving stations (pcs) c214
	Meteorological disaster prediction capacity c22	Storm warning accuracy rate c221
		Early warning of strong convective weather c222
		The accuracy rate of 24-h clear rain forecast c223
	Early warning information transmission capacity c23	The coverage rate of social units of meteorological warning information c231
		The coverage rate of meteorological early warning information broadcast media c232
		The coverage rate of meteorological early warning information social institutions c233
	Disaster-related information acquisition capacity c24	Two-way sharing rate of meteorological disaster information departments c241
		The rate of meteorological information personnel in place in villages (communities) c242
		Township (street) meteorological coordinators in place c243
Disaster relief capability c3	Social resource mobilization capacity c31	Insurance premium income c311
		Welfare Lottery Fund Expenditure c312
	Proactive disaster risk reduction capacity c32	Available rockets c321
		Available anti-aircraft guns c322
	Disaster relief security capacity c33	Number of beds in medical institutions per 10,000 people c331
		Local financial expenditure on medical and health care c332
		Local general public service expenditures per capita (10,000 yuan) c333

**Table 2:** Weight intervals of indicators at each level.

Primary Indicator	Weight Range	Secondary Indicator	Weight Range	Tertiary Indicator	Weight Range
c1	[0.3, 0.5]	c11	[0.3, 0.6]	c111	[0.5, 0.7]
				c112	[0.25, 0.4]
				c113	[0.25, 0.4]
		c12	[0.5, 0.7]	c121	[0.5, 1]
		c13	[0.2, 0.3]	c131	[0.5, 1]
c2	[0.4, 0.6]	c21	[0.3, 0.5]	c211	[0.25, 0.4]
				c212	[0.25, 0.4]
				c213	[0.25, 0.4]
				c214	[0.25, 0.4]
		c22	[0.4, 0.6]	c221	[0.4, 0.6]
				c222	[0.4, 0.6]
				c223	[0.2, 0.4]
		c23	[0.1, 0.2]	c231	[0.3, 0.5]
				c232	[0.4, 0.5]
				c233	[0.3, 0.5]
		c24	[0.2, 0.3]	c241	[0.25, 0.4]
				c242	[0.5, 0.7]
				c243	[0.25, 0.4]
c3	[0.3, 0.5]	c31	[0.5, 0.7]	c311	[0.5, 0.7]
				c312	[0.5, 0.7]
		c32	[0.3, 0.6]	c321	[0.5, 0.7]
				c322	[0.5, 0.7]
		c33	[0.2, 0.5]	c331	[0.25, 0.4]
				c332	[0.5, 0.7]
				c333	[0.25, 0.4]

**Table 3:** Generalized Shapley values for the three levels of indicators.

	$c_{211}$	$c_{212}$	$c_{213}$	$c_{214}$	$c_{221}$	$c_{222}$	$c_{223}$
$v\left(c\right)$	0.4	0.25	0.25	0.25	0.6	0.4	0.2
$\varphi_{c}^{sh}\left(g_{\lambda },C\right)$	0.354	0.215	0.282	0.215	0.517	0.327	0.156
	$c_{231}$	$c_{232}$	$c_{233}$	$c_{241}$	$c_{242}$	$c_{253}$	$c_{311}$
$v\left(c\right)$	0.5	0.4	0.3	0.25	0.5	0.4	0.5
$\varphi_{c}^{sh}\left(g_{\lambda },C\right)$	0.425	0.332	0.243	0.211	0.442	0.347	0.4
	$c_{312}$	$c_{321}$	$c_{322}$	$c_{331}$	$c_{332}$	$c_{333}$	
$v\left(c\right)$	0.7	0.7	0.5	0.4	0.5	0.25	
$\varphi_{c}^{sh}\left(g_{\lambda },C\right)$	0.6	0.6	0.4	0.347	0.442	0.211	

**Table 4:** Evaluation values of secondary indicators in 2014.

Province	$c_{11}$	$c_{12}$	$c_{13}$	$c_{21}$	$c_{22}$	$c_{23}$	$c_{24}$	$c_{31}$	$c_{32}$	$c_{33}$
Beijing	0.030	0.038	0.006	0.006	0.036	0.040	0.035	0.055	0.010	0.031
Tianjin	0.023	0.014	0.003	0.004	0.030	0.033	0.033	0.011	0.010	0.019
Hebei	0.031	0.042	0.025	0.033	0.102	0.030	0.030	0.040	0.033	0.039
Shanxi	0.040	0.015	0.008	0.031	0.029	0.023	0.030	0.018	0.022	0.026
Inner Mongolia	0.023	0.023	0.012	0.038	0.030	0.040	0.034	0.020	0.055	0.027
Liaoning	0.079	0.043	0.044	0.034	0.075	0.037	0.035	0.031	0.031	0.035
Jilin	0.026	0.027	0.040	0.022	0.032	0.029	0.026	0.010	0.034	0.025
Heilongjiang	0.032	0.030	0.032	0.037	0.028	0.034	0.030	0.019	0.096	0.027
Shanghai	0.026	0.021	0.001	0.011	0.032	0.040	0.035	0.037	0.005	0.027
Jiangsu	0.029	0.061	0.004	0.029	0.030	0.035	0.036	0.064	0.008	0.056
Zhejiang	0.029	0.084	0.053	0.052	0.031	0.034	0.027	0.061	0.000	0.040
Anhui	0.045	0.031	0.039	0.031	0.032	0.035	0.033	0.035	0.012	0.035
Fujian	0.044	0.032	0.024	0.036	0.030	0.032	0.035	0.027	0.000	0.028
Jiangxi	0.027	0.040	0.036	0.033	0.029	0.026	0.028	0.029	0.068	0.031
Shandong	0.032	0.061	0.026	0.040	0.031	0.035	0.031	0.066	0.004	0.054
Henan	0.030	0.037	0.050	0.057	0.056	0.030	0.034	0.047	0.052	0.053
Hubei	0.052	0.051	0.149	0.057	0.031	0.035	0.031	0.039	0.030	0.042
Hunan	0.035	0.038	0.059	0.042	0.033	0.035	0.033	0.036	0.029	0.041
Guangdong	0.023	0.084	0.053	0.034	0.035	0.036	0.035	0.083	0.006	0.067
Guangxi	0.034	0.019	0.080	0.038	0.028	0.033	0.035	0.021	0.018	0.033
Hainan	0.029	0.009	0.013	0.013	0.039	0.036	0.029	0.005	0.002	0.015
Chongqing	0.046	0.019	0.014	0.021	0.028	0.032	0.031	0.027	0.019	0.028
Sichuan	0.069	0.050	0.043	0.062	0.028	0.031	0.032	0.048	0.062	0.049
Guizhou	0.040	0.023	0.052	0.042	0.029	0.026	0.033	0.016	0.056	0.034
Yunnan	0.024	0.015	0.045	0.058	0.029	0.040	0.025	0.024	0.107	0.033
Tibet	0.019	0.000	0.004	0.028	0.028	0.027	0.035	0.013	0.029	0.016
Shaanxi	0.055	0.023	0.010	0.040	0.052	0.039	0.032	0.025	0.047	0.032
Gansu	0.045	0.023	0.013	0.035	0.031	0.027	0.034	0.016	0.037	0.026
Qinghai	0.027	0.005	0.038	0.025	0.028	0.043	0.027	0.006	0.025	0.018
Ningxia	0.030	0.008	0.003	0.014	0.031	0.031	0.034	0.004	0.014	0.015
Xinjiang	0.033	0.034	0.022	0.068	0.052	0.032	0.025	0.028	0.142	0.030

**Table 5:** Generalized Shapley values of secondary indicators.

	$c_{11}$	$c_{12}$	$c_{13}$	$c_{21}$	$c_{22}$	$c_{23}$	$c_{24}$	$c_{31}$	$c_{32}$	$c_{33}$
$v\left(c\right)$	0.3	0.7	0.2	0.5	0.4	0.2	0.3	0.5	0.3	0.5
$\varphi_{c}^{sh}\left(g_{\lambda },C\right)$	0.234	0.614	0.152	0.371	0.287	0.218	0.208	0.390	0.210	0.390

**Table 6:** Primary indicator evaluation values and comprehensive evaluation value (CEV) for 2014.

Province	$c_{1}$	$c_{2}$	$c_{3}$	CEV
Beijing	0.027	0.030	0.033	0.029
Tianjin	0.014	0.026	0.013	0.017
Hebei	0.031	0.053	0.037	0.038
Shanxi	0.019	0.028	0.022	0.022
Inner Mongolia	0.020	0.035	0.034	0.028
Liaoning	0.050	0.046	0.032	0.041
Jilin	0.029	0.026	0.022	0.025
Heilongjiang	0.031	0.032	0.049	0.036
Shanghai	0.018	0.030	0.022	0.023
Jiangsu	0.031	0.032	0.041	0.034
Zhejiang	0.056	0.037	0.034	0.041
Anhui	0.038	0.032	0.026	0.031
Fujian	0.033	0.032	0.017	0.026
Jiangxi	0.035	0.028	0.043	0.034
Shandong	0.036	0.034	0.040	0.035
Henan	0.039	0.045	0.050	0.043
Hubei	0.073	0.040	0.036	0.048
Hunan	0.042	0.035	0.034	0.036
Guangdong	0.056	0.034	0.050	0.045
Guangxi	0.041	0.033	0.024	0.031
Hainan	0.015	0.029	0.008	0.016
Chongqing	0.023	0.028	0.024	0.024
Sichuan	0.052	0.040	0.052	0.046
Guizhou	0.038	0.033	0.034	0.034
Yunnan	0.026	0.040	0.057	0.039
Tibet	0.006	0.029	0.019	0.017
Shaanxi	0.027	0.040	0.034	0.032
Gansu	0.025	0.031	0.026	0.027
Qinghai	0.025	0.032	0.015	0.023
Ningxia	0.012	0.028	0.010	0.016
Xinjiang	0.029	0.046	0.070	0.046

**Table 7:** CEV by province, 2014–2018.

Province	2014	2015	2016	2017	2018
Beijing	0.029	0.027	0.028	0.027	0.027
Tianjin	0.017	0.018	0.017	0.016	0.017
Hebei	0.038	0.033	0.032	0.031	0.031
Shanxi	0.022	0.023	0.022	0.022	0.026
Inner Mongolia	0.028	0.030	0.033	0.030	0.026
Liaoning	0.041	0.040	0.034	0.033	0.031
Jilin	0.025	0.027	0.031	0.031	0.030
Heilongjiang	0.036	0.035	0.035	0.036	0.034
Shanghai	0.023	0.024	0.028	0.025	0.022
Jiangsu	0.034	0.034	0.034	0.036	0.039
Zhejiang	0.041	0.042	0.041	0.042	0.043
Anhui	0.031	0.031	0.031	0.031	0.029
Fujian	0.026	0.027	0.029	0.029	0.027
Jiangxi	0.034	0.029	0.029	0.030	0.029
Shandong	0.035	0.042	0.040	0.041	0.041
Henan	0.043	0.041	0.037	0.040	0.040
Hubei	0.048	0.046	0.044	0.043	0.047
Hunan	0.036	0.039	0.038	0.039	0.036
Guangdong	0.045	0.045	0.051	0.049	0.052
Guangxi	0.031	0.031	0.031	0.032	0.035
Hainan	0.016	0.015	0.016	0.016	0.017
Chongqing	0.024	0.027	0.024	0.024	0.024
Sichuan	0.046	0.046	0.045	0.043	0.041
Guizhou	0.034	0.033	0.035	0.036	0.036
Yunnan	0.039	0.041	0.043	0.044	0.047
Tibet	0.017	0.016	0.016	0.017	0.017
Shaanxi	0.032	0.031	0.032	0.033	0.033
Gansu	0.027	0.027	0.029	0.029	0.030
Qinghai	0.023	0.023	0.025	0.028	0.026
Ningxia	0.016	0.017	0.016	0.016	0.017
Xinjiang	0.046	0.045	0.044	0.042	0.040

**Table 8:** Weights of entropy weight method.

Tertiary Indicators	2014	2015	2016	2017	2018
**c111**	0.1236	0.1311	0.1169	0.1051	0.0682
**c112**	0.0100	0.0078	0.0074	0.0039	0.0038
**c113**	0.0001	0.0001	0.0000	0.0000	0.0000
**c121**	0.0474	0.0519	0.0548	0.0599	0.0606
**c131**	0.0854	0.0894	0.1049	0.1036	0.1212
**c211**	0.0644	0.0688	0.0819	0.0876	0.0861
**c212**	0.0390	0.0433	0.0475	0.0470	0.0417
**c213**	0.0291	0.0326	0.0362	0.0395	0.0267
**c214**	0.0445	0.0471	0.0515	0.0559	0.0628
**c221**	0.0539	0.0614	0.0672	0.0729	0.0768
**c222**	0.0433	0.0477	0.0522	0.0566	0.0430
**c223**	0.0000	0.0054	0.0022	0.0015	0.0018
**c231**	0.0983	0.0939	0.0208	0.0029	0.0391
**c232**	0.0002	0.0004	0.0003	0.0004	0.0005
**c233**	0.0001	0.0003	0.0001	0.0000	0.0000
**c241**	0.0064	0.0012	0.0006	0.0005	0.0001
**c242**	0.0251	0.0113	0.0040	0.0037	0.0029
**c243**	0.1278	0.1304	0.1528	0.1540	0.1518
**c311**	0.1206	0.0952	0.1088	0.1043	0.1094
**c312**	0.0106	0.0044	0.0028	0.0021	0.0011
**c321**	0.0001	0.0001	0.0000	0.0000	0.0000
**c322**	0.0003	0.0000	0.0002	0.0002	0.0000
**c331**	0.0020	0.0020	0.0023	0.0026	0.0029
**c332**	0.0337	0.0368	0.0432	0.0478	0.0488
**c333**	0.0341	0.0375	0.0413	0.0481	0.0506

**Table 9:** Comparison of the weights of primary indicators.

Year	Methods	c1	c2	c3
2014	Interaction method	0.304	0.304	0.392
	Entropy weight TOPSIS method	0.267	0.312	0.422
2015	Interaction method	0.304	0.304	0.392
	Entropy weight TOPSIS method	0.280	0.306	0.414
2016	Interaction method	0.240	0.519	0.240
	Entropy weight TOPSIS method	0.284	0.238	0.478
2017	Interaction method	0.240	0.519	0.240
	Entropy weight TOPSIS method	0.272	0.236	0.491
2018	Interaction method	0.240	0.519	0.240
	Entropy weight TOPSIS method	0.254	0.255	0.491
